# Alpha-Synuclein Accumulation and Its Phosphorylation in the Enteric Nervous System of Patients Without Neurodegeneration: An Explorative Study

**DOI:** 10.3389/fnagi.2020.575481

**Published:** 2020-11-23

**Authors:** Lu-Lu Bu, Kai-Xun Huang, De-Zhi Zheng, Dan-Yu Lin, Ying Chen, Xiu-Na Jing, Yan-Ran Liang, En-Xiang Tao

**Affiliations:** ^1^Department of Neurology, Sun Yat-sen Memorial Hospital, Sun Yat-sen University, Guangzhou, China; ^2^Guangdong Provincial Key Laboratory of Malignant Tumor Epigenetics and Gene Regulation, Sun Yat-sen Memorial Hospital, Sun Yat-sen University, Guangzhou, China; ^3^Department of Neurology, The Eighth Affiliated Hospital, Sun Yat-sen University, Shenzhen, China

**Keywords:** α-Synuclein, α-synucleinopathies, enteric nervous system, non-neurodegenerative disorder, pathology

## Abstract

Alpha-synuclein (α-Syn) is widely distributed and involved in the regulation of the nervous system. The phosphorylation of α-Syn at serine 129 (p^Ser129^α-Syn) is known to be closely associated with α-Synucleinopathies, especially Parkinson's disease (PD). The present study aimed to explore the α-Syn accumulation and its phosphorylation in the enteric nervous system (ENS) in patients without neurodegeneration. Patients who underwent colorectal surgery for either malignant or benign tumors that were not suitable for endoscopic resection (*n* = 19) were recruited to obtain normal intestinal specimens, which were used to assess α-Syn immunoreactivity patterns using α-Syn and p^Ser129^α-Syn antibodies. Furthermore, the sub-location of α-Syn in neurons was identified by α-Syn/neurofilament double staining. Semi-quantitative counting was used to evaluate the expression of α-Syn and p^Ser129^α-Syn in the ENS. Positive staining of α-Syn was detected in all intestinal layers in patients with non-neurodegenerative diseases. There was no significant correlation between the distribution of α-Syn and age (*p* = 0.554) or tumor stage (*p* = 0.751). Positive staining for p^Ser129^α-Syn was only observed in the submucosa and myenteric plexus layers. The accumulation of p^Ser129^α-Syn increased with age. In addition, we found that the degenerative changes of the ENS were related to the degree of tumor malignancy (*p* = 0.022). The deposits of α-Syn were present in the ENS of patients with non-neurodegenerative disorders; particularly the age-dependent expression of p^Ser129^α-Syn in the submucosa and myenteric plexus. The current findings of α-Syn immunostaining in the ENS under near non-pathological conditions weaken the basis of using α-Syn pathology as a suitable hallmark to diagnose α-Synucleinopathies including PD. However, our data provided unique perspectives to study gastrointestinal dysfunction in non-neurodegenerative disorders. These findings provide new evidence to elucidate the neuropathological characteristics and α-Syn pathology pattern of the ENS in non-neurodegenerative conditions.

## Introduction

Alpha-synuclein (α-Syn) is widely distributed in the nervous system and is involved in the regulation of synaptic plasticity, as well as the packaging and trafficking of vesicles (Fortin et al., 2004; Wislet-Gendebien et al., 2006). The physiological role of α-Syn is often associated with the exocytotic and synaptic machinery controlling neurotransmitter release (Alegre-Abarrategui et al., 2019).

The complexity of α-Syn pathology mainly reflects its ability to form a broad range of structures, its diverse post-translational modification status, and its ability to associate with both lipid and protein chaperones (Alegre-Abarrategui et al., 2019). The monomeric form of α-Syn is generally considered to be non-pathogenic and has profound implications for neuronal physiology. Under certain circumstances, α-Syn would fold from its natural state into pathogenic forms comprising oligomers and polymers, and could converge into a fibrous filament, called the fiber precursor (preformed fibrils, PFFs). Insoluble fibrils of phosphorylated α-Syn (p-α-Syn) have also been implicated in inducing the pathological progress of neurodegenerative disorders (Peng et al., 2018; Bieri et al., 2019), of which phosphorylation at serine 129 (p^Ser129^α-Syn) has been shown to be the most abundant form in intracellular inclusion bodies in the brain of patients with PD (Oueslati, 2016). The term α-Synucleinopathies refers to neurodegenerative disorders that share the key pathological feature of neuronal loss accompanied by the presence of α-Syn inclusions, including Parkinson's disease (PD), dementia with Lewy bodies (DLB), and multiple system atrophy (MSA). Besides these typical α-Synucleinopathies, patients with Alzheimer's disease (AD) also show an overlap of α-Syn, Aβ plaques, and tau tangle pathologies (Lippa et al., 1998).

Pathologically, α-Syn spreads between neurons like prions, which induces more monomeric α-Syn misfolding or over-modification, and is then deposited in neurons to form Lewy bodies (LBs), leading to neurotoxicity (Mao et al., 2016; Lim and Lee, 2017; Urrea et al., 2018). LB pathology in α-Synucleinopathies is not confined to the central nervous system (CNS). The involvement of the peripheral autonomic nervous system was first observed more than 50 years ago (den and Bethlem, 1960). The enteric nervous system (ENS) is composed of neurons embedded in the gastrointestinal tract and is known as the “second brain” (Rao and Gershon, 2016; Chalazonitis and Rao, 2018). Neurons and enteric glial cells in the ENS form the gastrointestinal ganglia, also known as the nerve plexus, mainly comprising the submucosal and myenteric plexus (Sprenger et al., 2015). Mounting evidence indicates that α-Syn is present in the ENS of patients with PD and related disorders (Lee et al., 2018). As reported by Del Tredici and Braak (2016), α-Syn pathology could commence in the ENS and then spread to the brain to participate in the pathogenesis and progression of PD (Braak et al., 2003; Breen et al., 2019). In a post-mortem study, α-Syn was identified in both patients with PD and in pathologically defined cases of sporadic DLB, but it was not detected in the controls (Annerino et al., 2012). However, more recent evidence has indicated that α-Syn is a normal constituent of the ENS and thus exists in healthy controls as well as patients with PD (Bottner et al., 2012; Gold et al., 2013; Antunes et al., 2016; Shin et al., 2017). Although considerable progress has been made in determining the distribution pattern of α-Syn pathology, variation in the status of α-Syn in healthy people still remains.

Few data are available on the normal spatial expression profile and distribution pattern of α-Syn in the ENS. Therefore, this study aimed to explore the status of α-Syn accumulation and its phosphorylation in the human ENS of patients with non-neurodegenerative diseases, i.e., approximating to non-pathological conditions. We employed the normal intestine tissues of 19 patients that underwent colorectal surgery for malignant or benign tumors who were not suitable for endoscopic resection to investigate α-Syn pathology in the normal ENS. The results showed that deposits of α-Syn and p^Ser129^α-Syn in the ENS could be detected in people with non-neurodegenerative disorders. The results revealed the neuropathological characteristics and α-Syn pathology pattern of ENS in non-neurodegenerative conditions and might indicate that the α-Syn pathology of the ENS represents physiological deposition.

## Materials and Methods

### Participants

This study was approved by the Ethics Committee of Sun Yat-sen Memorial Hospital, Sun Yat-sen University, Guangzhou, China (Approval number: 2015 Lun Bei NO. 50). All the involved patients [11 male and 8 female, aged 39–84 years (mean age, 65)] attended Sun Yat-sen Memorial Hospital. Written informed consent for this research was obtained from all individuals prior to surgery. Intestinal specimens were obtained from patients who underwent colorectal surgery for malignant or benign tumors that were not suitable for endoscopic resection. A 2 cm-wide sample of the intestinal tract was taken as the initial specimen within a safe distance (10 cm) from the tumor (Bottner et al., 2012). Specimens were subdivided into Ileum (Il), right colon (Rc), left colon (Lc), and rectum (Re) groups.

All patients underwent a complete pre-operative neurological examination to rule out neurodegenerative disease-related symptoms, particularly cognitive impairment and PD-related symptoms. Patients with a history of neurological or psychiatric disorders were excluded. All patients were assessed for the presence of PD using the diagnostic criteria for PD issued by the International Parkinson and movement disorder society in 2015 (Postuma et al., 2015). The TNM (Tumor-Node-Metastasis) stage of each subject was assessed according to the Classification of Malignant Tumor, 8th Edition, published by the Union for International Cancer Control in 2016. Then, the patients were staged according to their prognosis (0–IV), together with the pathological results. There were eight tumor sub-stages, comprising stages 0, I, IIa–IIb, IIIa–IIIc, and IVa, respectively. A score of 0 was indicative of a tumor free status, although evidence of inflammation was present.

### Immunohistochemistry and Quantitative Analysis

Immunostaining of neurofilaments (NFs), α-Syn and pathologic α-Syn were employed to assess their distribution patterns. For immunohistochemical staining, the initial tissue was immersed in 4% neutral formalin solution for fixation within 1 h after tissue extraction. All specimens were fixed for more than 48 h. Then, a 1.5 cm × 1.5 cm full-thickness intestinal sample was selected for paraffin embedding and preparation. Continuous sectioning was performed, and the thickness of each section was 4 μm.

After baking (60°C, 1.5–2 h), xylene dewaxing, and gradient alcohol (100–90–75%) dehydration, antigen retrieval of the samples on the slides was performed in a microwave oven with high fire for 10 min and medium-high fire for 10 min, using sodium citrate solution (0.01 mol/L, pH 6.0). Then, the sections were blocked using an endogenous peroxidase blocker (10 min) and 5% goat serum (15 min). They were then incubated with primary antibodies (anti-α-Syn-monoclonal antibody, 1:500, ab138501, Abcam, Cambridge, MA, USA; anti-neurofilament-polyclonal antibody, 1:500, ab235991, Abcam; and an anti-phospho-α-Syn (Ser129) antibody, 1:500, MABN826, Millipore, Billerica, MA, USA) at 4°C overnight, followed by incubation with a biotin-conjugated secondary antibody (1:3,000; BA-1000, Vector Laboratories, Burlingame, CA, USA) for 15 min at room temperature (RT) and horseradish peroxidase (HRP)-labeled streptavidin (1:1,000, PK-6100; Vector Laboratories) for 10 min at RT. After reaction with 3,3′-Diaminobenzidine (DAB) peroxidase substrate (SK-4100, Vector Laboratories), the slides were stained with hematoxylin, dried, and sealed with neutral resin. Detailed steps of immunohistochemistry are provided in the Supplementary Material. All other reagents were acquired from Sigma-Aldrich (Shanghai, China).

The immunohistochemically-stained sections were imaged using a Nikon eclipse 80 microscope and counted as follows: 1. The NF positive neuron count. The number of positive neurons in the myenteric plexus were counted at high magnification (20×) and the NF statistic was determined by the number of neurons observed and divided by the number of visual fields observed; 2. α-Syn expression. A semi-quantitative counting method was used to record the frequency of α-Syn expression, as 0- absence, 1- sparse (≤ 1/4 positive staining area), and 2- small amount (≤ 2/4 positive staining area), 3- common (≤ 3/4 positive staining area), 4- rich (> 3/4 positive staining area) according to criteria from a previous study (Lebouvier et al., 2010). For this study, the positive staining area referred to the proportion of the positively stained nerve plexus in the entire enteric nerve plexus of a complete specimen, rather than taking an average of the positive rates of several random fields; 3. p^Ser129^α-Syn. In a high power field of view, starting from one end of the myenteric plexus of the specimen, we obtained the visual fields of the specimen covering the entire myenteric plexus without overlap. Five visual fields across the plexus were assigned at random for counting, and the ratio of p^Ser129^α-Syn positive neurons was generated from the mean of the number of p^Ser129^α-Syn positive neurons divided by the total number of neurons across the five inspected fields was calculated.

### Statistical Analysis

The rostro caudal gradient of LBs in the gastrointestinal nervous system prompted us to divide the samples into four groups according to the sampling site, namely, the ileum group (Il); right colon group (Rc); left colon group (Lc), including the descending colon and sigmoid colon; and the rectum group (Re). This was done to exclude the influence of different sampling sites and to verify the rostro caudal gradient.

All experiments were performed in triplicate. Data are expressed as the mean ± SD. Data normality was tested using the Kolmogorov-Smirnov test. Differences between means were evaluated using a one-way analysis of variance (ANOVA) test followed by a homogeneity test using IBM SPSS Statistics 20 software (IBM Corp., Armonk, NY, USA). A least significant difference (LSD)-*t-*test was used for multiple comparisons, including the inter-group comparison of NF and p^Ser129^α-Syn. For semi-quantitative analysis, the Kruskal–Wallis test was used to compare multiple samples between groups. The correlations were analyzed using Pearson correlation, except for the correlation analysis involving α-Syn and tumor staging, which was analyzed using the Spearman correlation test. The statistical significance was set at ^*^P < 0.05, ^**^P < 0.01, and ^***^P < 0.001).

## Results

### Characteristics of the Population

A total of 11 male and 8 female patients, aged between 39 and 84 years old, consented to participate and were enrolled in this study. They underwent colorectal surgery for either malignant tumors of the intestinal tract or benign tumors and were not suitable for endoscopic resection. The basic demographic data of patients in this study are shown in Table 1. Clinical information, including sex, age, admission and discharge diagnosis, pathological diagnosis, tumor stage, levels of tumor markers were obtained and are presented in the Supplementary Materials.

**Table 1:** Demographic and clinical data of patients underwent colorectal surgery.

Age	31–40 (*n =* 1)	41–50 (*n =* 2)	51–60 (*n =* 6)	61–70 (*n =* 4)	71–80 (*n =* 4)	81–90 (*n =* 2)
Specimens	Ileum (*n =* 3)	Right colon (*n =* 4)		Left colon (*n =* 4)		Rectum (*n =* 8)
Tumor stage	0 (*n =* 1)	I (*n =* 3)	II (*n =* 6)	III (*n =* 7)		IV (*n =* 2)

### Distribution of Neurons in the ENS of Patients With Non-neurodegenerative Diseases

**Figure 1:**
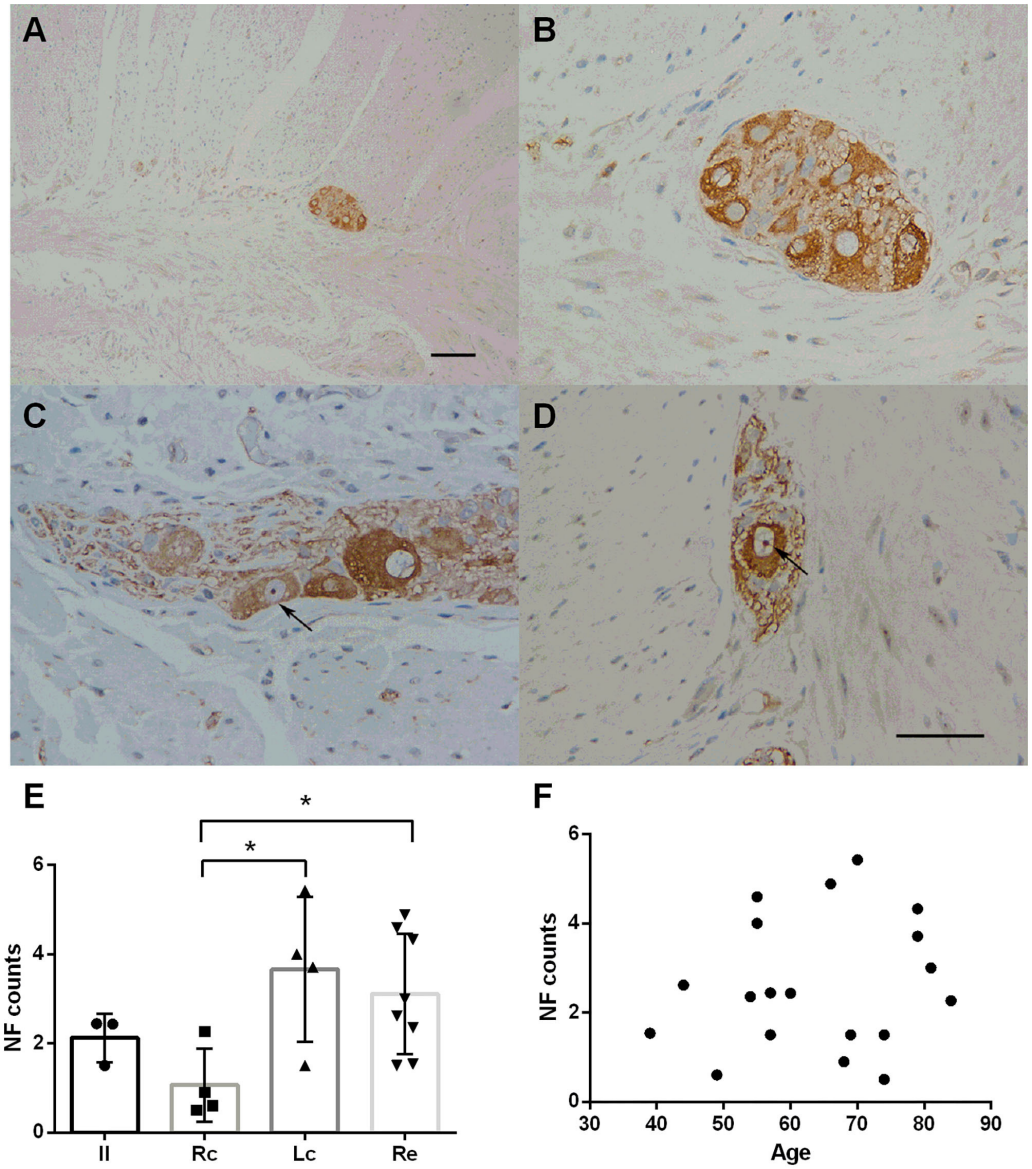
Immunostaining of neurofilaments (NFs) in the myenteric plexus. **(A–D)** Immunostaining of NFs in the myenteric plexus. Representative image of the myenteric nerve plexus located between the two muscle layers (longitudinal and circular muscles), comprised of many nerve plexus sections on the cut surface **(A)**. Fine staining of NF-positive profiles (brown color) could be seen in the perikaryon of neurons in the myenteric plexus. The nuclei of the neurons were lightly stained and some nucleoli could be seen (black arrows in **C,D**). Hematoxylin counterstain, scale bars = 100 μm **(A)**, 50 μm **(B–D)**. **(E)** Quantitative analysis of the NF-positive neuron count in the myenteric plexus. The count is subdivided into the ileum (Il), right colon (RC), left colon (LC), and rectum (RE). Inter-group differences are reported as per one-way ANOVA (*F* = 3.564, *df* = 18, *p* = 0.040) followed by an LSD-t *post-hoc* test (RC group 1.07 ± 0.82 *vs*. the LC group 3.66 ± 0.62, *p* = 0.010; RC group 1.07 ± 0.82 *vs*. the RE group 3.11 ± 1.35, *p* = 0. 017). Data are shown as NF positive neurons per visual field for individual cases and the group mean ± SD is also shown. **(F)** Scatter plot of the NF-positive neuron count within the myenteric plexus with age. No significant correlation was observed (Pearson correlation, *p* = 0.499). * = *p* < 0.05.

NF staining was identified throughout the intestinal tract (100%). However, neurons in the mucosa and submucosa were too scattered to count and rate. The myenteric plexus was selected for comparison among the groups. Representative immunostaining of NFs in the myenteric plexus is shown in Figure 1. Fine staining of NFs (brown color) could be seen in the perikaryon of neurons in the myenteric plexus (black arrows in Figures 1B,D). No statistically significant difference was found in age distribution between the groups (*p* = 0.822). There were inter-group differences in the NF counts among the Rc, Lc, and Re groups (one-way ANOVA, *F* = 3.564, *df* = 18, *p* = 0.040, followed by a LSD-t *post-hoc* test: The Rc group 1.07 ± 0.82 *vs*. the Lc group 3.66 ± 0.62, *p* = 0.010; the Rc group 1.07 ± 0.82 *vs*. the Re group 3.11 ± 1.35, *p* = 0.017) (Figure 1E). The neuronal distribution in the myenteric plexus was not correlated with age (Pearson correlation, *p* = 0.281) (Figure 1F).

### Distribution and Expression of α-Syn and Pathological α-Syn in the ENS

**Figure 2:**
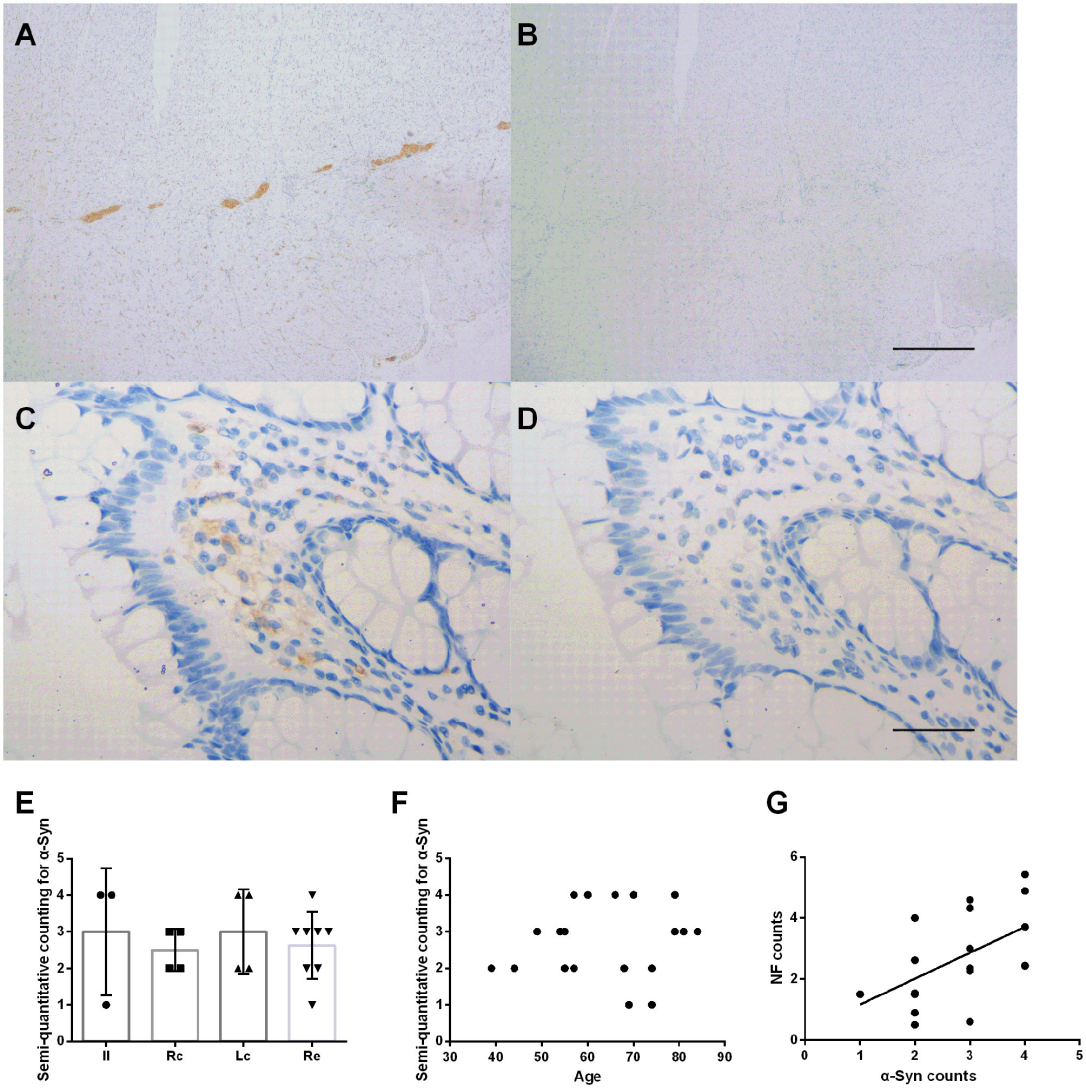
Characteristic of α-Syn immunoreactive signals in the myenteric plexus. **(A–D)**. Representative image of immunostaining for α-Syn in the myenteric plexus. α-Syn immunoreactive signals (brown color) in the myenteric plexus **(A)**, and the mucosa **(C)**. **(B,D)** Are negative controls. Hematoxylin counterstain, scale bars = 500 μm **(A,B)**, 50 μm **(C,D)**. **(E)** Specimens were subdivided into the ileum (Il), right colon (Rc), left colon (Lc), and rectum (Re). Semi-quantitative analysis of α-Syn immunoreactive signals among the groups in the myenteric plexus (Kruskal–Wallis test, *p* = 0.812). Data shown as mean± SD. **(F)** Scatter plot of the semi-quantitative analysis of α-Syn immunoreactive signals within the myenteric plexus with age. No significant correlation was observed (Pearson correlation, *p* = 0.554). **(G)** Scatter plot of the NF-positive neuron count within the myenteric plexus with α-Syn showed a positive correlation (Pearson correlation, *R* = 0.592, *p* = 0.008).

**Figure 3:**
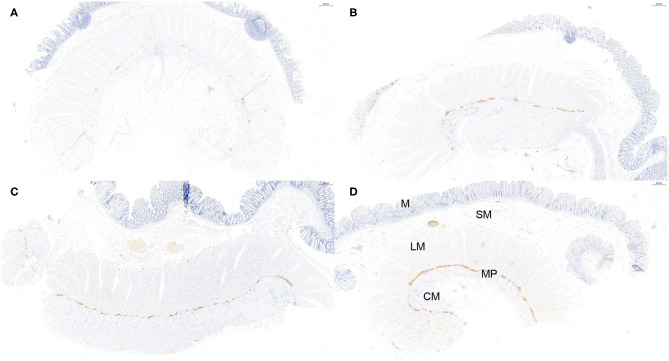
Semi-quantitative counting for α-Syn expression in the myenteric plexus. All layers of the intestine were shown. From top to bottom: The mucosa (M), submucosa (SM), longitudinal muscularis (LM), myenteric plexus (MP), and circular muscularis (CM), respectively. The expression of α-Syn was defined as 1- sparse (≤ 1/4 positive staining area) **(A)**, 2- small amount (≤ 2/4 positive staining area) **(B)**, 3-common (≤ 3/4 positive staining area) **(C)**, and 4-rich (> 3/4 positive staining area) **(D)**, according to a previous study. The “positive” stained area here refers to the length of the positively stained myenteric plexus as a proportion of the total length of the specimen. Hematoxylin counterstain, scale bars = 500 μm.

Immunohistochemical staining showed that the deposition of α-Syn could be detected in all specimens of the submucosa, muscularis propria, myenteric plexus, and part of the mucosa (12/19, 63%) (Figure 2). Figure 2 shows representative images of α-Syn in the myenteric plexus (Figure 2A), and the mucosa (Figure 2C) obtained via immunostaining. Non-specific staining interference was excluded. The Kruskal–Wallis test showed that there was no significant difference in the expression of α-Syn among groups in the myenteric plexus (Kruskal–Wallis test, *p* = 0.812) (Figure 2E). The deposition of α-Syn showed no significant correlation with age in the myenteric plexus (Pearson correlation, *p* = 0.554) (Figure 2F), whereas it positively correlated with the count of neurons (Pearson correlation, *R* = 0.592, *p* = 0.008) (Figure 2G). Semi-quantitative counting for α-Syn expression in the intestinal tract was defined according to a previous study, as shown in Figure 3.

**Figure 4:**
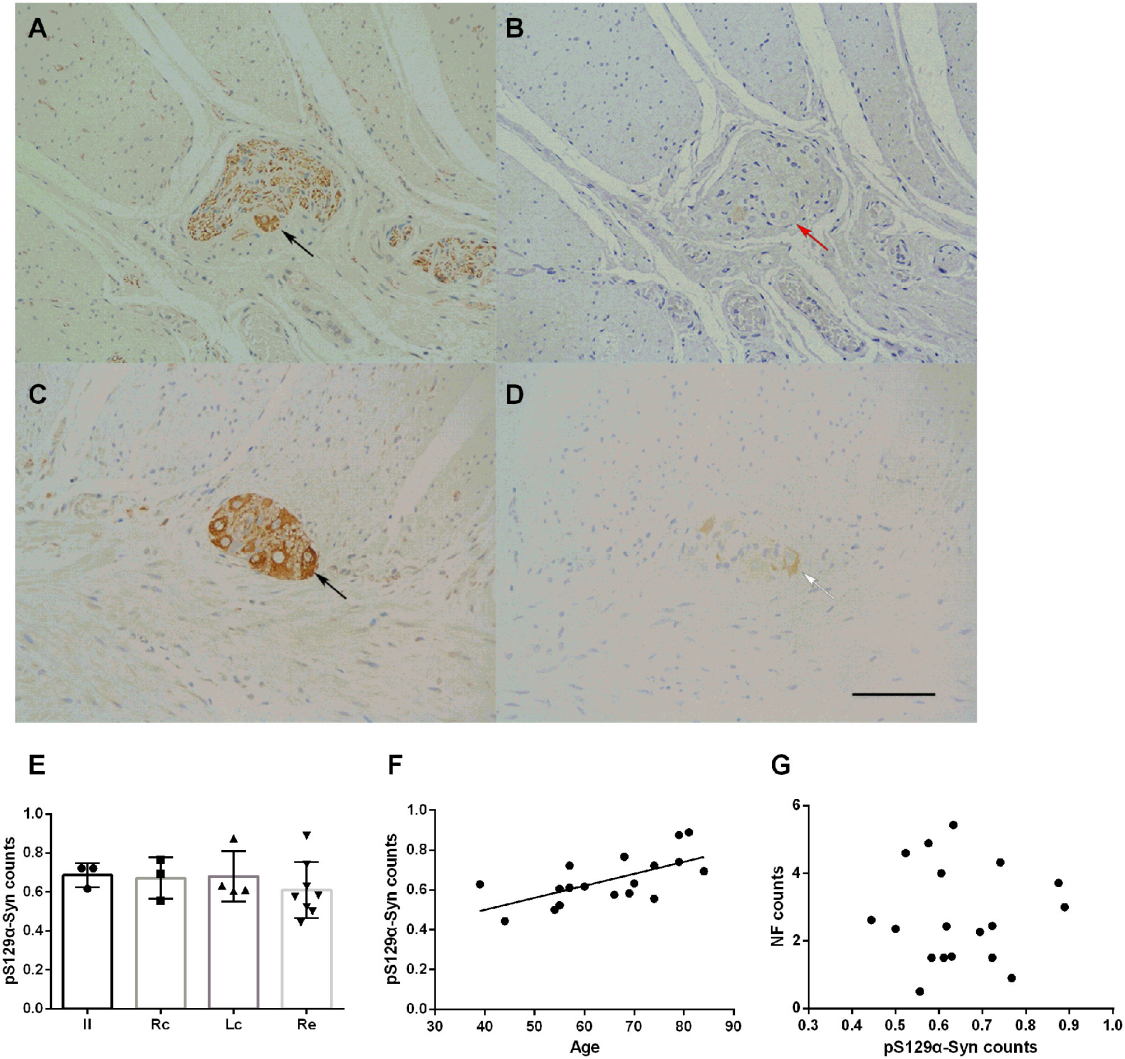
Characteristic of p^S129^α-Syn immunoreactive signals in the myenteric plexus **(A–D)**. Representative image of NF immunoreactivity in the myenteric plexus (black arrows in **A,C**). p^S129^α-Syn immunoreactivity in the human myenteric plexus **(B,D)** (brown color). Co-localization of NFs and p^S129^α-Syn (white arrow in **D**). Not all neurons with positive NF staining have p^S129^α-Syn deposition (red arrow in **B**). Hematoxylin counterstain, bars =50 μm. **(E)** Quantitative analysis of the p^S129^α-Syn-positive count in the myenteric plexus. Specimens were subdivided into the Ileum(Il), right colon (RC), left colon (LC), and rectum (RE). Inter-group differences are reported as per one-way ANOVA (*F* = 0.452, *df* = 17, *p* = 0.720) followed by LSD-t *post-hoc* test. Data are shown as p^S129^α-Syn positive neurons per visual field for individual cases and the group mean ± SD is also shown. **(F)** Scatter plot of the p^S129^α-Syn-positive neuron count within the myenteric plexus with age showed a positive correlation (Pearson correlation, *R* = 0.643, *p* = 0.004). **(G)** Scatter plot of p^S129^α-Syn immunoreactive signals within the myenteric plexus with the NF-positive neuron count. No significant correlation was observed (Pearson correlation, *p* = 0.958).

The deposition of p^S129^α-Syn was observed only in the submucosa and the myenteric plexus and was detected in the cytoplasm of neurons. We used serial section staining to analyze co-localization. The representative distribution and expression of pathological α-Syn were observed, as shown in Figures 4A–D. Not all neurons in the myenteric plexus had p^S129^α-Syn deposits. No significant difference was observed in the deposition of p^S129^α-Syn between groups (one-way ANOVA, *F* = 0.452, *df* = 17, *p* = 0.720) (Figure 4E). Correlation analysis indicated that there was an age-dependent deposition of p^S129^α-Syn in the myenteric plexus (*R* = 0.643, *p* = 0.004) (Figure 4F), suggesting that the deposition of p^S129^α-Syn in the ENS myenteric plexus increased with age. However, the deposition of p^S129^α-Syn and the count of neurons in the myenteric plexus showed no correlation (*p* = 0.958) (Figure 4G).

### α-Syn Pathology of the ENS and Its Correlation With Tumor Malignancy

**Figure 5:**
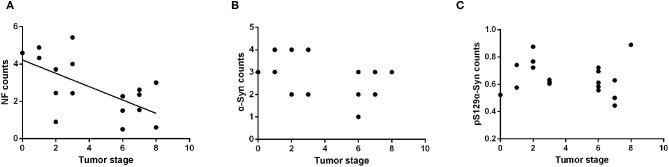
Correlation analysis between the α-Syn pathology of the ENS and tumor malignancy. **(A)** Scatter plot of p^S129^α-Syn immunoreactive signals within the myenteric plexus with tumor malignancy; a negative correlation was observed (Pearson correlation, *R* = −0.522, *p* = 0.022). **(B)** Scatter plot of α-Syn immunoreactive signals within the myenteric plexus with tumor malignancy; no significant correlation was observed (Spearman correlation, *p* = 0.095). **(C)** Scatter plot of p^S129^α-Syn immunoreactive signals within the myenteric plexus with tumor malignancy; no significant correlation was observed (Pearson correlation, *p* = 0.566).

All subjects included in this study were patients with primary intestinal tumors; therefore, excluding the neoplastic effect was necessary to analyze the influence of tumor malignancy on the α-Syn pathology. The Kruskal–Wallis test showed no statistically significant differences in the tumor stage among the four groups (*p* = 0.751). Spearman correlation analysis suggested a negative correlation between the tumor stage and the NF counts in the myenteric plexus of the ENS (Pearson correlation, *R* = −0.522, *p* = 0.022) (Figure 5A). There was no significant correlation between the deposition of α-Syn and tumor stage in the myenteric plexus (Spearman correlation, *p* = 0.095) (Figure 5B). Furthermore, the deposition of p^Ser129^α-Syn did not correlate with the tumor stage (Pearson correlation, *p* = 0.566) (Figure 5C). Taken together, these findings suggested that α-Syn pathology in ENS might not be affected by tumor malignancy.

## Discussion

In the present study, we explored the distribution of α-Syn and its phosphorylation modification in the non-neurodegenerative intestinal tracts. We employed full-thickness normal intestinal tissues from 19 patients with non-neurodegenerative diseases to explore the levels of α-Syn and phosphorylated α-Syn via immunohistochemistry staining. We found that in the intestinal tissues of patients without neurodegeneration, accumulation of α-Syn and p^Ser129^α-Syn were detected within the submucosal and myenteric plexus, as well as in enteric nerve fibers. This evidence argues against the role of ENS immunostaining of α-Syn as a diagnostic hallmark for α-Synucleinopathies and related neurodegenerative disorders. Our findings indicated that the level of p^Ser129^α-Syn was age-dependent in the myenteric plexus, suggesting that the accumulation of α-Syn in the ENS might be a physiological change.

The presence of α-Syn inclusions is a common hallmark of α-Synucleinopathies. However, why it would accumulate in the ENS remains unknown. Without a clear understanding of the function of α-Syn and why it would accumulate in a nerve cell, we cannot unravel the pathophysiology of diseases such as PD. Previous studies using antibodies against α-Syn and p-α-Syn in the ENS of patients with neurodegenerative diseases have yielded conflicting results. Reports have suggested the accumulation of α-Syn in the ENS in animals and humans under both physiological and pathological states. A previous report investigating α-Syn immunoreactivity in biopsies of the distal sigmoid colon in 10 patients suggested that immunostaining of nerve fibers of the colonic submucosa was observed in all PD biopsies, but not the controls (Shannon et al., 2012). In addition, α-Syn was identified both in patients with PD and in pathologically-defined cases of sporadic DLB, but not in the controls (Annerino et al., 2012). α-Syn deposits could be observed in the ENS in MSA (Pouclet et al., 2012). In contrast, however, other studies found that α-Syn was a normal constituent of the ENS and was thus present both in healthy controls and participants with PD (Bottner et al., 2012; Gold et al., 2013; Antunes et al., 2016; Shin et al., 2017). Aggregated α-Syn and p^Ser129^α-Syn were present in individuals with and without PD (Visanji et al., 2015). In a human post-mortem study on elderly subjects without neurological diseases, α-Syn immunoreactivity was observed in the brainstem, olfactory nerves, spinal cord, and the peripheral nervous system (Bloch et al., 2006). There are several possible reasons for the inconsistencies between the results of past studies on α-Syn in the ENS. These include differences in the sites and depths of biopsies, and the antibodies and histological techniques used. Previous studies proved a rostro-caudal gradient of α-Syn expression, with greater density of expression in rostral portions as compared to the more caudal portions of the gastrointestinal tract (Beach et al., 2010; Gelpi et al., 2014). It suggested that localization of biopsies along the gastrointestinal tract and the difference in intestinal staining intensity might be critical factors that could be standardized in studies of enteric α-Syn immunohistochemistry, and may be responsible for previous discrepancies in results. These findings raise the question of whether α-Syn immunostaining in the ENS holds promise as a diagnostic hallmark and represents the pathological state.

Our study is consistent with previous immunohistochemical studies on intestinal biopsies, in which α-Syn pathology in the ENS is not a proper indicator of α-Synucleinopathies. The presence of α-Syn in the human ENS appears to be a normal finding. In contrast to native α-Syn immunoreactivity, which remained uniform regardless of the patients' age, we found that the level of p^Ser129^α-Syn was age-dependent in the myenteric plexus of the ENS. Normal development and aging could be major contributing factors to α-Syn pathology. Correlation analysis showed that the counts of neurons in the myenteric plexus correlated positively with the accumulation of α-Syn (*p* = 0.008). It is also supported the hypothesis that the accumulation of α-Syn is a physiological phenomenon.

Data from previous human studies have shown that p^Ser129^α-Syn occurs in the adult brain physiologically (Muntane et al., 2012). Another study found that the presence of LBs was not associated with the severity of neurodegeneration in the CNS (Schulz-Schaeffer, 2010). We found that p^Ser129^α-Syn levels increased with age, and previous studies indicated that the pathology of p^Ser129^α-Syn was not limited to PD, and was implicated in several neurodegenerative diseases (Paleologou et al., 2010; Vaikath et al., 2019). Therefore, we speculated that p^Ser129^α-Syn might reflect the aging process to a certain extent, rather than just neurodegeneration. These changes can also be found in patients without neurodegenerative disorders; therefore, these changes might not be regarded as a hallmark in patients with neurodegenerative disorders, particularly PD.

Correlation analysis showed that there was no significant correlation between the distribution of p^Ser129^α-Syn and age or tumor stage. While the presence of p^Ser129^α-Syn was observed only in the submucosa and myenteric plexus, the age-dependent pattern indicated that this pathological change represented constant accumulation with age. Additionally, we found that the degenerative changes of the ENS were related to the degree of tumor malignancy.

The significance of this research was to determine the importance of α-Syn pathology as an intestinal hallmark for neurodegenerative disorders. We showed that positive staining of α-Syn was also detected in normal intestinal specimens of the human ENS. The pathological change of p^Ser129^α-Syn was observed to constantly accumulate with age in the ENS and might not be strictly regarded as a pathological correlation. In addition, there was no significant correlation between the α-Syn pathology of the ENS and tumor malignancy. These results suggested that α-Syn pathology in the ENS is not suitable as a hallmark of α-Synucleinopathies such as PD.

There are several limitations to this study. The recruitment challenges arising from the need to obtain full-thickness intestinal specimens meant that patients with neurodegenerative disorders and healthy individuals are lacking in the current study. Further studies are needed to compare these groups and to investigate the role of α-Syn pathology in the ENS. We should also determine whether this deposit is pathogenic, related to the digestive tract, or caused by extrapyramidal symptoms. The present study was insufficient to show the different forms of α-Syn, such as the prefibrillar forms and oligomers. Furthermore, only a small group of tumor patients were enrolled in this study. Our results may be limited by the sample size and the lack of intestinal tissue from patients with α-Synucleinopathies, including PD. In addition, the differences in intestinal staining intensity (under different conditions) between non-neurodegenerative cases and neurodegenerative cases might also cause different results. However, in the current study, we only focused on exploring the α-Syn status in the nearly normal ENS. Further studies involving larger samples and patients with neurodegeneration patients are required to provide stronger evidence for our conclusions.

## Conclusion

Overall, we found that deposits of α-Syn in the ENS were presented in people with non-neurodegenerative disorders in a physiological state. In particular, the age-dependent appearance of p^Ser129^α-Syn was observed in the submucosa and myenteric plexus. This finding weakens the basis and reliability of gastrointestinal pathology as an auxiliary diagnosis for α-Synucleinopathies, including PD.

## Data Availability Statement

The original contributions presented in the study are included in the article/Supplementary Materials, further inquiries can be directed to the corresponding author/s.

## Ethics Statement

The studies involving human participants were reviewed and approved by Ethics Committee of Sun Yat-sen Memorial Hospital, Sun Yat-sen University, Guangzhou, China. The patients/participants provided their written informed consent to participate in this study.

## Author Contributions

E-XT, Y-RL, and X-NJ designed the experiments and critically revised the manuscript. K-XH and D-ZZ performed the experiments. L-LB and D-YL collected patient information and specimens. L-LB, YC, and X-NJ analyzed and interpreted the data. L-LB and X-NJ wrote the manuscript. All authors read and approved the final manuscript. All authors contributed to the article and approved the submitted version.

## Conflict of Interest

The authors declare that the research was conducted in the absence of any commercial or financial relationships that could be construed as a potential conflict of interest.
