# Supplementary material for: Alpha-Synuclein Accumulation and Its Phosphorylation in the Enteric Nervous System of Patients Without Neurodegeneration: An Explorative Study
Source: Front Aging Neurosci. 2020 Nov 23;12:575481. doi: 10.3389/fnagi.2020.575481 (PMC7719782; doi:10.3389/fnagi.2020.575481)
Supplement: Supplementary file 1 [file Data_Sheet_2.DOCX]

PROTOCOL for Immunohistochemistry

STEP1: Baked slices, Baked slices at 60 degrees for 1.5 hours;

STEP2: Dewaxing, Xylene twice for 20min each;

STEP3: Hydration, Gradient alcohol(100%-90%-75%) for 10min each;

STEP4: PBS washing three times for 3min each(use shaking table in low speed);

STEP5: At the third time of the previous step, we start to preheat sodium citrate on medium-high heat;

STEP6: Antigen retrieval, use microwave oven, high fire for 10min, medium-high fire for 10min;

STEP7: PBS washing three times for 4min each(in low speed);

STEP8: Add endogenous peroxidase blocker and incubate for 10 minutes;

STEP9: PBS washing three times for 4min each(in low speed);

STEP10: Add animal non-immune serum (sheep) and block for 15 minutes;

STEP11: Incubate the primary antibody at 4 degrees overnight;

STEP12: PBS washing three times for 5min each(in normal speed);

STEP13: Incubate the secondary antibody (biotin-labeled goat anti-mouse/rabbit IgG) for 15 minutes;

STEP14: PBS washing three times for 5min each;

STEP15: Use streptavidin-peroxidase and incubate for 10 min;

STEP16: PBS washing three times for 5min each;

STEP17: Add DAB and observe under the microscope immediately. When a light brown coloration is observed in the background, quickly put it in PBS for washing to avoid too dark DAB coloration;

STEP18: PBS washing three times for 5min each;

STEP19: Hematoxylin staining (average 14-16s), and observe under the microscope whether the staining is too deep;

STEP20: After dyeing, PBS washing for 4min for the first time, then tap water washing twice for 4min each;

STEP21: Bake in a 60° oven for 30 minutes, and then cover the film with neutral resin and observe it under a microscope.
